# Improved depiction of hemodynamics in intracranial aneurysms by 4D flow MRI at 7T compared to 3T

**DOI:** 10.1186/1532-429X-15-S1-W12

**Published:** 2013-01-30

**Authors:** P van Ooij, R Kleinloog, JJ Zwanenburg, F Visser, P Luijten, AJ Barker, M Markl, A Nederveen, CB Majoie, L Regli, G Rinkel, B Verweij

**Affiliations:** 1 Radiology, Feinberg School of Medicine, Northwestern University, Chicago, IL, USA; 2 Neurology and Neurosurgery, University Medical Center Utrecht, Utrecht, Netherlands; 3 Department of Radiology, Image Sciences Institute, University Medical Center Utrecht, Utrecht, Netherlands; 4 Department of Radiology, Academic Medical Center, Amsterdam, Netherlands; 5 Department of Neurosurgery, University Hospital Zürich, Zürich, Switzerland

## Background

Intracranial aneurysms are life threatening conditions and occur in 3-6% of the population. The annual rupture rate is approximately 2% associated with significant morbidity and mortality. Hemodynamic information obtained by 4D flow MRI may contribute to the assessment of rupture risk of intracranial aneurysms. However, the small sizes of intracranial aneurysms require high spatial resolution to accurately capture complex intra-aneurysmal flow. Increasing spatial resolution, however, decreases signal-to-noise ratio (SNR) and thus velocity-to-noise ratio in flow images. Performing 4D flow at 7T has high potential to overcome low SNR limits and enable higher spatial resolution in 4D flow acquisitions. In this study 4D flow in intracranial aneurysms at 7T was compared with 4D flow at 3T.

## Methods

Retrospectively gated 4D flow measurements were performed in two aneurysms on a 3T MR system (Philips Healthcare, Best, Netherlands) using an 8-channel coil and in two different aneurysms on a 7T whole body system (Philips Healthcare, Cleveland, OH, USA) with a 32-channel coil (Nova Medical, Wilmington, MA, USA). Scan parameters 3T: voxel size: 0.8 x 0.8 x 0.8 mm; FOV: 200 x 200 x 20 mm; TE/TR: 5.7/8.5 ms; FA: 10°; cardiac phases: 10. Scan parameters 7T: voxel size: 0.5 x 0.5 x 0.5 mm, FOV: 180 x 180 x 20 mm; TE/TR: 4.1/8.6 ms; FA: 20°; cardiac phases: 6; Both scanners: venc: 100 x 100 x 100 cm/s; SENSE factor: 3. Scan time at both scanners was kept constant (15-20 min) by exchanging temporal for spatial resolution. Phase images were corrected for background offset errors by subtraction of the average phase in a static region of interest (amygdala). The lumen in both scans was semi-automatically segmented at all cardiac phases and in every slice in the phase contrast magnitude images using a level set evolution algorithm. All aneurysms were located in the middle cerebral artery; the sizes of the aneurysms are given in table 1.

**Table 1:** Size (length x width x height) of the aneurysms

Ane	Field	Size (l x w x h)
1	3T	12.6 x 7.3 x 9.1
		
2		13.0 x 7.9 x11.3

3	7T	7.1 x 7.1 x 10.8
		
4		8.7 x 10.4 x 6.9

## Results

**Figure 1:**
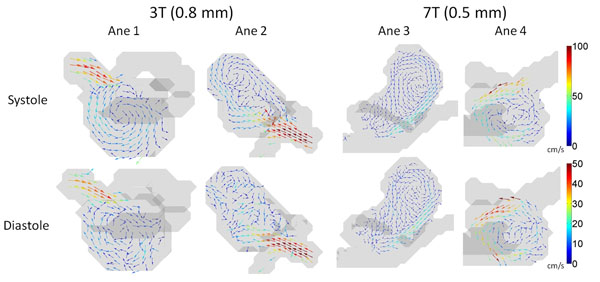
Flow patterns in characteristic slices in two aneurysms acquired at 3T and two aneurysms at 7T

Figure 1 illustrates characteristic flow patterns acquired at 3T (left) and 7T (right) during systole and diastole for all aneurysms included in the study. Results derived from 4D flow MRI at 3T for aneurysms 1 and 2 show irregular or incoherent vortex profiles in diastole. In contrast, 4D flow MRI at 7T in aneurysms 3 and 4 resulted in a more coherent depiction of flow patterns and vortices.

## Conclusions

It was not possible to perform SNR quantification by subtraction of images at different cardiac phases, since the lower amount of cardiac phases acquired at 7T would underestimate signal and overestimate noise compared to 3T. However, from figure 1 it is clear that the SNR in diastole was improved at 7T compared to 3T due to more coherent vortices observed in the aneurysms. The use of a 32-channel coil at 7T may have increased the SNR further. The results of this feasibility study indicate that 4D flow at 7T in aneurysms has the potential to acquire slow moving flow fields with better accuracy at even higher spatial resolutions compared to 3T.

